# Microbial Processes and Microbial Communities in the Water Column of the Polar Meromictic Lake Bol’shie Khruslomeny at the White Sea Coast

**DOI:** 10.3389/fmicb.2020.01945

**Published:** 2020-08-11

**Authors:** Alexander S. Savvichev, Vitaly V. Kadnikov, Igor I. Rusanov, Alexey V. Beletsky, Elena D. Krasnova, Dmitry A. Voronov, Anna Yu. Kallistova, Elena F. Veslopolova, Elena E. Zakharova, Nataliya M. Kokryatskaya, Galina N. Losyuk, Nikolai A. Demidenko, Nikolai A. Belyaev, Pavel A. Sigalevich, Andrey V. Mardanov, Nikolai V. Ravin, Nikolay V. Pimenov

**Affiliations:** ^1^Winogradsky Institute of Microbiology, Research Center of Biotechnology, Russian Academy of Sciences, Moscow, Russia; ^2^Institute of Bioengineering, Research Center of Biotechnology, Russian Academy of Sciences, Moscow, Russia; ^3^Faculty of Biology, Lomonosov Moscow State University, Moscow, Russia; ^4^N. Laverov Federal Center for Integrated Arctic Research, Ural Branch, Russian Academy of Sciences, Moscow, Russia; ^5^Zubov State Oceanographic Institute, Moscow, Russia; ^6^Shirshov Institute of Oceanology, Russian Academy of Sciences, Moscow, Russia

**Keywords:** carbon cycle, sulfur cycle, meromictic lakes, microbial communities, White Sea, anoxygenic phototrophic bacteria, *Chlorobium*, carbon isotope fractionation

## Abstract

Microbiological, molecular ecological, biogeochemical, and isotope geochemical research was carried out at the polar Lake Bol’shie Khruslomeny at the coast of the Kandalaksha Bay, White Sea in March and September 2017. The uppermost mixolimnion was oxic, with low salinity (3–5%). The lower chemocline layer was brown-green colored, with very high content of particulate organic matter (up to 11.8 mg C L^–1^). The lowermost monimolimnion had marine salinity (22–24%) and very high concentrations of sulfide (up to 18 mmol L^–1^) and CH_4_ (up to 1.8 mmol L^–1^). In the chemocline, total microbial abundance and the rate of anoxygenic photosynthesis were 8.8 × 10^6^ cells mL^–1^ and 34.4 μmol C L^–1^ day^–1^, respectively. Both in March and September, sulfate reduction rate increased with depth, peaking (up to 0.6–1.1 μmol S L^–1^ day^–1^) in the lower chemocline. Methane oxidation rates in the chemocline were up to 85 and 180 nmol CH_4_ L^–1^ day^–1^ in March and September, respectively; stimulation of this process by light was observed in September. The percentages of cyanobacteria and methanotrophs in the layer where light-induced methane oxidation occurred were similar, ∼2.5% of the microbial community. Light did not stimulate methane oxidation in deeper layers. The carbon isotope composition of particulate organic matter (δ^13^C-Corg), dissolved carbonates (δ^13^C-DIC), and methane (δ^13^C- CH_4_) indicated high microbial activity in the chemocline. Analysis of the 16S rRNA gene sequences revealed predominance of Cyanobium cyanobacteria (order Synechococcales) in the mixolimnion. Green sulfur bacteria *Chlorobium phaeovibrioides* capable of anoxygenic photosynthesis constituted ∼20% of the chemocline community both in March and in September. *Methyloprofundus gammaptoteobacteria* (family Methylomonaceae) were present in the upper chemocline, where active methane oxidation occurred. During winter, cyanobacteria were less abundant in the chemocline, while methanotrophs occurred in higher horizons, including the under-ice layer. Chemolithotrophic gammaproteobacteria of the genus Thiomicrorhabdus, oxidizing reduced sulfur compounds at low oxygen concentrations, were revealed in the chemocline in March. Both in March and September archaea constituted up to 50% of all microorganisms in the hypolimnion. The percentage of putative methanogens in the archaeal community was low, and they occurred mainly in near-bottom horizons.

## Introduction

Meromictic (permanently stratified) lakes are formations in which microbial geochemical activity is most pronounced (Overmann et al., 1991; Crowe et al., 2014; Walter et al., 2014). The highest rates of microbial processes occur in the zone where oxic and anoxic (euxinic, oxygen-free, sulfide-enriched) water layers contact. This zone is termed a chemocline (Overmann et al., 1991), pycnocline (Galand et al., 2012), a transitory zone (Schmidtova et al., 2009), or an oxic–anoxic interface (Vetriani et al., 2003). Among the natural compounds acting as oxidizers, oxygen is the most energetically advantageous electron acceptor for heterotrophic organisms consuming organic matter (Wang and Van Cappellen, 1996). Microorganisms oxidize reduced sulfur, iron, and manganese compounds (Canfield et al., 1993), as well as methane (Eller et al., 2005), primarily using oxygen. Oxygen respiration of heterotrophic and chemolithotrophic microorganisms may occur at very low ambient oxygen levels, which are below 120 nM, the threshold for conventional oxygen sensors (Stolper et al., 2010). The processes occurring at micromolar oxygen concentrations have not been studied until recently (Brand et al., 2016).

Prokaryotes are known to be the only organisms capable of methane oxidation in aquatic ecosystems under both oxic and anoxic conditions (Reeburgh, 2007; Bastviken et al., 2008). In the absence of oxygen and presence of sulfate, methane is consumed mainly due to sulfate-dependent anaerobic methane oxidation (AOM), which is responsible for the oxidation of over 90% methane in anoxic zones of marine environments (Strous and Jetten, 2004; Knittel and Boetius, 2009). In the water column of meromictic lakes, microbial methane oxidation is known to occur, apart from the oxic zone, also at the oxic-anoxic interface (Schubert et al., 2010), as well as in the anoxic zone, where oxygen can not be detected (Blees et al., 2014; Milucka et al., 2015). Light was shown to stimulate methane oxidation in anoxic lake water (Milucka et al., 2015; Oswald et al., 2015; Kallistova et al., 2018). This may indicate that oxygen used by methanotrophs for methane oxidation may be produced via oxygenic photosynthesis. While the minimum light requirement for oxygenic phototrophs is considered to be ∼2 μEm^–2^ s^–1^ (Raven et al., 2000), it may in fact be significantly lower. Gibson (1985) reported the minimal photon flow of 0.1 to 0.5 μE m^–2^ s^–1^ for freshwater and 0.02 μE m^–2^s^–1^ for marine environments. Moreover, anoxygenic phototrophs, e.g., *Chlorobiaceae*, may carry out anoxygenic photosynthesis at illumination levels below 0.01 μEm^–2^ s^–1^ (Raven et al., 2000).

In the chemocline zone of meromictic lakes, a complex multilayer microbial community develops, which carries out conversion of carbon, sulfur, iron, and manganese compounds (Kuznetsov, 1970; Ivanov et al., 2001; Pimenov et al., 2003; Savvichev et al., 2004, 2018; Wand et al., 2006; Crowe et al., 2010; Rogozin et al., 2010, 2017; Galand et al., 2012).

Meromictic lakes may act as models for investigation of microbial biogeochemical processes occurring at the oxic-anoxic interface (Koeksoy et al., 2016; Lambrecht et al., 2019). Moreover, investigation of the composition and function of microbial communities involved in the methane biogeochemical cycle is also important for the understanding of climatic changes (Singh et al., 2010; Boetius, 2019). Among diverse meromictic basins, those located in coastal areas and to some extent connected to the sea (constantly of periocically) are of special interest. Several lakes of such type at the Cape Kindo coast (Kandalaksha Bay, White Sea) have recently attracted attention of researchers: Lakes Kislo-Sladkoe, Trekhtsvetnoe, and Elovoe. The coastal stratified lakes of the Kandalaksha shore, White Sea, are unique in origin and have very young age. They developed due to gradual separation from the sea caused by rapid rising of the land (∼40 cm in the last 100 years), which started with deglaciation and is presently going on (Krasnova et al., 2015). Moreover, this area is characterized by highly indented coastline and abundance of islands (Krasnova et al., 2015). In the course of land rising and separation of these bays from the sea, the upper water layer becomes desalinated, and marine biota is replaced by the freshwater one. Applied value of research on microbial communities and microbial processes in the basins separated from the major sea basin stems from the necessity to predict the possibility of sulfide contamination in artificially closed sea areas resulting from construction of dams, piers, tidal power stations, etc. (Velinsky and Fogel, 1999; Savvichev et al., 2017).

Investigation of these basins revealed a pronounced peak of light ÑÎ_2_ fixation in the chemocline zone, which was associated with activity of anoxygenic phototrophic bacteria (Savvichev et al., 2014). Predominant organisms in this layer were green-colored green sulfur bacteria (GSB) *Chlorobium chlorovibrioides*, while brown-colored GSB *Chlorobium phaeovibrioides*, as well as purple sulfur bacteria *Thiocapsa rosea* and *Thiorhodococcus kakinadensis* and non-sulfur purple bacteria *Rhodovulum sulfidophilum*, predominated in the lower horizon (Lunina et al., 2013). The pronounced red layer in the chemocline of Lake Kislo-Sladkoe was shown to contain high numbers of cryptophyte algae of the genus *Rhodomonas* (Cryptophyta, Pirenomonadaceae) (Krasnova et al., 2014). These basins are characterized by intense sulfate reduction in the chemocline and hypolimnion, as well as by involvement of photo- and chemoautotrophic bacteria in production of organic matter with unusual carbon isotope composition (Savvichev et al., 2014). The patterns of microbial processes in meromictic lakes depend significantly on their topographic location and genesis. The composition and functional activity of microbial communities depend on a number of parameters: temperature, salt composition, trophic status, depth of light penetration, etc.

Lake Bol’shie Khruslomeny (66° 42′ 59″ N, 32° 51′ 30″ E) is a meromictic basin of marine genesis. This small lake with indented coastline and the highest depth of 18.5 m is located on Olenii Island (Kandalaksha Bay, White Sea at the latitude of the Polar circle, 23 km from the White Sea Biological Station (WSBS) of the Moscow State University (Krasnova et al., 2015). The upper 2 m layer with low salinity is subject to wind mixing; salinity of the lower layers is higher. Seawater penetrates into the lake via a boulder dam in its southern part during syzygy tides and especially strong winds. Only ∼100 years ago, this lake was a regular sea gulf. A stratified water column developed during this short period of isolation from the sea. This evolution resulted in changes in the composition of the microbial community and in the scale of microbial processes. Establishing the present-day state of the microbial community and microbial processes in this rapidly evolving basin was the goal of the present work.

The goal of the present work was to determine the parameters of the microbial community in the water column of the meromictic Lake B. Khruslomeny using molecular genetic approaches, as well as the rates of microbial processes (autotrophic and heterotrophic CO_2_ fixation, sulfate reduction, methanogenesis, and methane oxidation), and to determine the carbon isotope composition of particulate organic matter, dissolved methane, and bicarbonate.

## Materials and Methods

### Sampling

**FIGURE 1:**
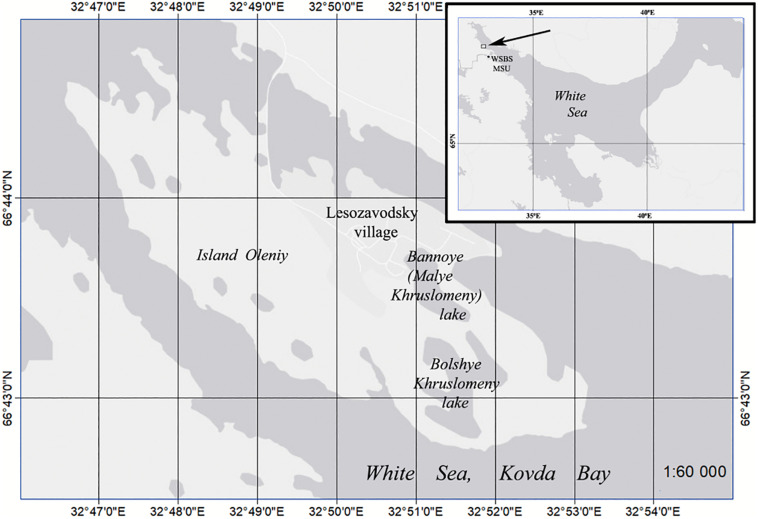
The polar meromictic Bol’shie Khruslomeny Lake (Kandalaksha Bay coast, White Sea). WSBS MSU, White Sea Biological Station, Moscow State University. The map was composed using https://yandex.ru/maps.

The work at the lake B. Khruslomeny (Figure 1) was carried out in March and September, 2017.

Water samples were collected at the point of the greatest depth (18.5 m). The samples were taken from various depths using a silicon rubber tube attached to a gaged line and a GP1352 Whale Premium Submersible Pump (Ireland). The samples were dispensed into 30-mL glass vials, sealed with gas-tight rubber stoppers avoiding air bubbles, and closed with perforated aluminum caps.

Illuminance was measured with an AR813A Luxmeter (China), with the registering unit modified for underwater use and mounted on a gaged cable line. The temperature and concentration of dissolved oxygen were measured with a WTW^©^ 340iA HANNA HI8314F (Wensoket, RI, United States) portable ionometer with temperature compensation; a combination electrode was used for pH measurement. Specific conductivity was determined with a HANNA HI8733 (Wensoket, RI, United States) portable conductometer.

Methane concentration in the water samples was determined by the headspace method (phase equilibrium degassing; McAuliffe, 1971). Methane was measured on a Kristall-2000-M gas chromatograph (Chromatec, Russia) equipped with a flame ionization detector. Sulfate and chloride were measured on a Staier ion chromatograph (Russia) after distillation and concentrating. Particulate organic carbon (POC) was determined as described previously (Vetrov et al., 2015). Sulfide was measured with *N,N*-dimethyl-*p*-phenylenediamine on an Ekspert-303 photometer (Russia). Three samples were used to obtain average values. Statistical processing of the results was carried out using MS Excel 2000.

For assessment of total microbial abundance = total microbial number (MA = TMN) and microbial biomass, water samples in glass vials were fixed with glutaraldehyde at the final concentration of 2%. Fixed samples (5–10 mL) were filtered through black polycarbonate 0.2-μm filters (Millipore, United States). The filters were stained with acridine orange (2 mg/mL) (Hobbie et al., 1977) and examined under an Olympus BX 41 epifluorescence microscope equipped with an Image Scope Color (M) visualization system. The cells were enumerated in 20 fields of view.

### Radiotracer Analysis

The rates of microbial processes: light and dark CO_2_ assimilation (LCA and DCA), sulfate reduction (SR), methanogenesis (MG), and methane oxidation (MO) were determined by radiotracer analysis using NaH^14^CO_3_, ^14^CH_4_, and Na_2_^35^SO_4_. LCA and DCA rates for each horizon were determined using one darkened vial and two transparent ones, to which 0.2 mL (20 μCi) of a NaH^14^CO_3_ solution was added. DCMU [3-(3,4-dichlorophenyl)-1,1-dimethylurea,10^–7^ Mm] was used as a selective inhibitor of oxygenic photosynthesis (Legendre et al., 1983). The vials were attached to a nylon cord and suspended from an ice station in winter or from a buoy in summer. After incubation (24 h) the samples were fixed with 1 mL of diluted HCl and filtered through 0.2-μm membranes. Production of oxygenic photosynthesis was calculated as the difference between the total and anoxygenic photosynthesis (the transparent vial with DCMU).

To determine the rates of other processes in the samples, incubation was also carried out *in situ.* After incubation, the samples were fixed with 1 mL of 0.1 M KOH. The samples were then analyzed in the laboratory as was described previously (Savvichev et al., 2018). Radioactivity of the products of the studied microbial processes was measured using a TRI-Carb TR scintillation counter (Packard, United States). For determination of the LCA and DCA rates, ^14^C-CO_2_ both in bacterial cells and in the extracellular dissolved organic matter was accounted for. The confidence intervals for the LCA, DCA, MO, and SR values varied from 10 to 40%.

### Carbon Isotope Analysis

For determination of the carbon isotope composition of suspended organic matter (δ^13^C-C_org_), water samples were filtered through calcined 47-mm GF/F glass fiber filters. The filters were then dried at 60°C. The filtrate was used to determine the carbon isotope composition of dissolved bicarbonate. Dried suspension on the GF/F filters was oxidized to CO_2_ by high-temperature incineration (560°C) in the presence of copper oxide as a catalyst. Mineral carbon (dissolved inorganic carbon = DIC, HCO_3_^–^ + CO_3_^2–^) was converted to BaCO_3_, from which CO_2_ was produced by fusion of barium carbonate with tin salts at 560°C. The value of δ^13^C characterizing the carbon isotope composition was determined on a Delta Plus mass spectrometer (Thermo Electron Corporation, Germany), using a PDB-calibrated standard, and calculated according to the known equation:

(1) δC13=[(C13)/(C12)]/sample[(C13)/(C12)]-standard1)×1000%

where [(^13^C)/(^12^C)]_sample_/[(^13^C)/(^12^C)]_standard_ are the ratios of occurrence of ^12^C and ^13^C atoms in the sample and in the standard, respectively. The international PDB standard used has the isotope occurrence ration (^13^C)/(^12^C) of 0.001172 (Craig, 1957). For methane, δ^13^C-CH_4_ was measured on a TRACE GC gas chromatograph (Thermo Fisher Scientific, United States) coupled to a Delta Plus mass spectrometer. The error of the δ^13^C measurements did not exceed ±0.1%.

### DNA Extraction, Sequencing and Read-Centric Analysis

Water samples from depths of 0.5, 1.5, 2.5, 3.25, 3.75, 4.25, 4.75, 5.25, 10, 15, and 18 (March) and from 0.5, 2, 3, 3.25, 3.75, 4, 4.25, 4.5, 4.75, 5.25, 7, 10, 15, and 18 m (September) were collected by overflowing into 0.5-L plastic bottles and sealed, avoiding gas bubbles. Microbial cells from a whole volume of each water sample (0.5 L) were concentrated on 0.2-μm filters (CA membrane filter, Sartorius) on the day of sampling. The filters were homogenized by trituration with liquid nitrogen, and the preparation of metagenomic DNA was isolated by a method based on lysis of the cells followed by treatment with a detergent.

PCR amplification of the 16S ribosomal RNA gene fragments containing the V3–V4 variable regions was carried out using the universal primers PRK341F (5′- CCTACGGGRBGCASCAG -3′) and PRK806R (5′- GGACTACYVGGGTATCTAAT -3′) (Yu et al., 2005). PCR fragments were then sequenced on a GS FLX genome analyzer (Roche) according to the Titanium protocol using the GS FLX Titanium Sequencing Kit XL+. Creation of the library, its amplification, and sequencing were carried out according to the relevant Roche protocols.

Reads starting with the forward primer were selected and trimmed to the same length of 250 bp using Mothur v.1.35.1 (Schloss et al., 2009). All the subsequent OTU analysis was done with Usearch v.11 (Edgar, 2010). Low-quality reads were filtered (fastq_maxee = 1.00) and high-quality reads were clustered into OTUs at 97% identity level. At the clustering stage chimera and singleton sequences were removed by the Usearch algorithm. Then all reads, including low-quality ones and singletons, were mapped to OTU representative sequences at 97% global identity level to determine OTU size for each sample. OTU taxonomic identification was performed using the SINA online alignment and classification platform and the Silva v. 1.2.11 database with default parameters (Pruesse et al., 2012; Quast et al., 2013), and searching for close sequences in GenBank using the BLASTN protocol. When a sequence with more than 95% similarity with the 16S rRNA gene of the described microorganism was detected, OTU was assigned to the corresponding genus. A total of 2177 OTU were classified, which included 196,564 sequences of 16S rRNA genes.

## Results

### Hydrological Characterization of Lake B. Khruslomeny

**FIGURE 2:**
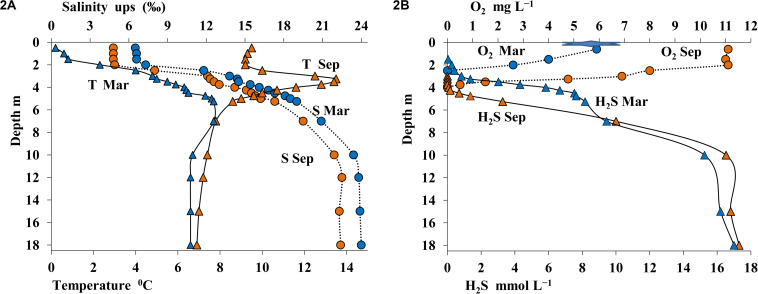
Physicochemical conditions in the water column of Lake B. Khruslomeny in March (blue) and September 2017 (orange): temperature,°C and salinity, ups **(A)** and concentrations of sulfide, mmol L^– 1^ and oxygen, mg L^– 1^ **(B)**.

Sampling was carried out twice: from open water in September and from ice in March. The thermohaline structure of Lake B. Khruslomeny was rather stable, with seasonal changes occurring mostly in the upper layer with low salinity. In winter, water temperature immediately below the ice was positive, albeit low (0.1–0.8°C), but at 2.0 m it already reached 2.3°C, and then slightly increased with depth (Figure 2A). In September the temperature of the upper layer was 9.5°C, with a well-pronounced warm layer (up to 13.5°C) observed in the chemocline horizons. Below the chemocline the temperature decreased to 7°C. In respect of salinity, three layers were revealed. The upper one (to 2-m depth), which was wind-mixed during the summer season, was desalinated (5–7%). Salinity increased sharply in the intermediate layer (2–4 m). In the hypolimnion salinity was as high as 22–24%.

**FIGURE 3:**
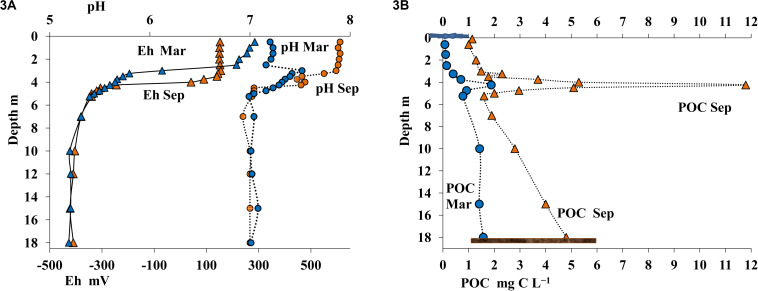
Values of pH and Eh **(A)** and content of particulate organic carbon (POC, mg C L^– 1^) **(B)** in the water column of Lake B. Khruslomeny in March (blue) and September (orange).

The desalinated upper layer contained dissolved oxygen. In September this oxygen-saturated layer reached the depth of 2 m, with oxygen concentration decreasing sharply in deeper layers; oxygen was detected by our instruments (with detection limit of 10 μmol O_2_ L^–1^) as low as at 3.75 m (Figure 2B). In winter oxygen concentrations were lower, and it was not detected below 2.5 m. The sulfide layer was located immediately below the oxic layer. Sulfide concentration in summer and in winter remained relatively stable. HS^–^ concentration in the chemocline increased sharply with depth (to 0.5-4 mmol L^–1^) and reached 18 mmol L^–1^ in the monimolimnion (Figure 2B). The pH and Eh values in the oxic layer exhibited pronounced seasonal differences. In the upper layer, pH was 7.8–7.9 in September and 7.2 in March. In March, pH peaked in the 3.0–3.25-m layer. Below the chemocline, pH and Eh values in summer and winter remained almost the same (pH = 7.0–7.1; Figure 3A).

The content of particulate organic carbon (POC) in September in the upper freshwater layer was ∼0.9–1.0 mg C L^–1^ (Figure 3B), which was more than five times higher than POC content in the White Sea water, but was common in freshwater reservoirs of the White Sea basin. POC content increased more than twofold in the intermediate saline layer. An extremely high POC level of 11.8 mg C L^–1^ was observed in September in the chemocline (4.25 m). This POC value was at least an order of magnitude higher than the concentrations observed in non-stratified basins. In the lower anoxic horizons below the 4.25-m layer, average POC levels were also more than twice higher than in the upper layers. In March, POC concentrations in all horizons were significantly lower than in September, reaching 0.075–0.14 mg C L^–1^ in the oxic zone, up to 1.880 mg C L^–1^ in the chemocline, and 0.780–1.570 mg C L^–1^ in the near-bottom water.

### Microbial Processes in the Water Column

#### Microbial Abundance and Biomass

**FIGURE 4:**
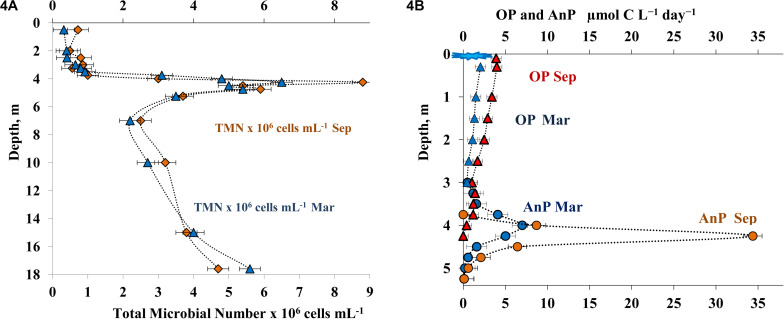
Total microbial numbers = microbial anbundance (TMN, 10^6^cells mL^– 1^) **(A)** and the rates of oxygenic photosynthesis (OP μmol C L^– 1^ day^– 1^) and anoxygenic photosynthesis (AnP μmol C L^– 1^ day^– 1^) **(B)** in the water column of Lake B. Khruslomeny in March (blue) and September (orange).

Microscopy of stained samples revealed heterogeneous distribution of microorganisms in the water column (Figure 4A). In both the summer and winter seasons, total microbial abundance (TMA) in the oxic layer was 0.2-0.8 × 10^6^ cells mL^–1^, which indicated the oligotrophic-mesotrophic state of the lake. At both seasons studied, TMA in the chemocline (4.25 m) was high, up to 8.8 × 10^6^ cells mL^–1^ in September and up to 7.6 × 10^6^ cells mL^–1^ in March. Below this peak, TMA decreased, but remained relatively high (2.5-5.4 × 10^6^ cells mL^–1^). Bacterial cell volume in the water column varied from 0.3 to 0.6 μm^3^, with 0.4-0.5 μm^3^ in the chemocline and 0.3-0.4 μm^3^ in the hypolimnion. The calculated microbial biomass was 40-80 μg C L^–1^ in the oxic layer, up to 880 μg C L^–1^ in the chemocline layer, and 180-360 μg C L^–1^ in the hypolimnion.

#### Production of Oxygenic and Anoxygenic Photosynthesis, Dark CO_2_ Assimilation (DCA)

Meromictic lakes, including Lake B. Khruslomeny, are characterized by two types of photosynthesis: oxygenic photosynthesis (OP), carried out in the upper water layer by cyanobacteria and eukaryotic algae, and anoxygenic photosynthesis (AnP), carried out in the anoxic zone by anoxygenic phototrophic bacteria (APB). In September, relatively low OP rates of 1.0-3.8 μmol C L^–1^ day^–1^ were observed in the oxic layer (0–3.75 m) (Figure 4B). The rate of oxygenic photosynthesis decreased significantly at the lower border of the oxic layer. At the same time, a pronounced AnP peak (up to 34.4 μmol C L^–1^ day^–1^) was revealed in the upper part of the sulfide-containing layer, at the horizons of 4.25 and 4.5 m. The rate of photosynthesis decreased rapidly with depth, reaching almost zero at 5.25 m. During the spring sampling (ice-covered period), OP rate was somewhat lower than in autumn (up to 2 μmol C L^–1^ day^–1^, Figure 4B) and was restricted to a narrow horizon below the ice. Significant rates of photosynthesis in winter indicated the possibility of light-induced methane oxidation. The relevant experiments were carried out in September (see below). The peak of anoxygenic photosynthesis occurred at this period in the 3.75-4.0 m horizon and was more shallow than in autumn. The highest AnP rate in March was 7.0 μmol C L^–1^ day^–1^.

**FIGURE 5:**
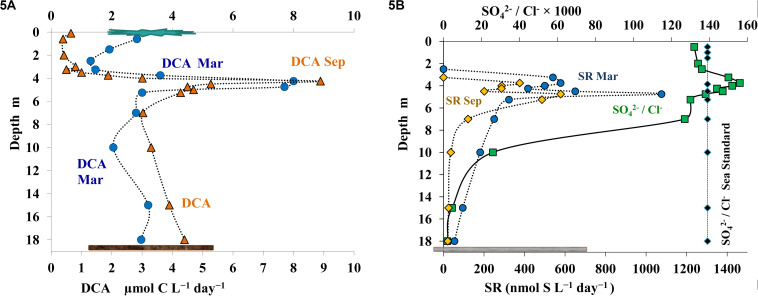
Rates of dark carbon assimilation (DCA, μmol C L^– 1^ day^– 1^) **(A)** and of sulfate reduction (SR) in March (blue) and September (orange), and the ratio of sulfate and chloride ions (SO_4_^2–^ /Cl^–^ × 1000) **(B)** in the water column of Lake B. Khruslomeny (green). Seawater standard (black) corresponds to SO_4_^2–^ = 2.649; Cl^–^ = 18.980 g/kg; SO_4_^2–^ /Cl^–^ × 1000 = 139.6.

Dark CO_2_ assimilation (DCA) is a summarized parameter including both the rate of heterotrophic carboxylation and autotrophic chemosynthetic CO_2_ assimilation (Alonso-Saez et al., 2010). In September (Figure 5A), DCA was low in the upper mixed layer (0.4-0.65 μmol C L^–1^ day^–1^), increased sharply in the chemocline, reaching the maximum at 4.25 m (8.89 μmol C L^–1^ day^–1^), and then decreasing with depth to 2.9-4.5 μmol C L^–1^ day^–1^ (Figure 5A). In March, high DCA was detected in the layer immediately below the ice (2.83 μmol C L^–1^ day^–1^), with the DCA peak of 8.0 μmol C L^–1^ day^–1^ occurring at 4.25 m, similar to the summer season; however, unlike summer, DCA remained high at 4.75 m (7.7 μmol C L^–1^ day^–1^).

#### Bacterial Sulfate Reduction

Radiotracer analysis showed first indications of sulfate reduction in March and September at the depths of 3.25 and 3.75 m, respectively, which corresponded to the upper horizon of the chemocline zone (Figure 5B). Both in summer and in winter sulfate reduction rates increased with depth and peaked (up to 0.6-1.1 μmol S L^–1^ day^–1^) in the lower chemocline (4.75 m). Below 5.25 m, sulfate reduction rates decreased to 0.25 μmol S L^–1^ day^–1^ at 7.0 m and 0.02–0.05 μmol S L^–1^ day^–1^ in the near-bottom water layer. Sulfate concentrations are an indirect indicator of the rates of microbial processes of the sulfur cycle (both oxidative and reductive). In marine basins with a constant ratio of the major salts, absolute sulfate concentrations may be used for the purpose. In stratified basins with salinity gradients in the water column, using sulfate share, expressed as a sulfate-chloride coefficient (SO_4_^2–^/Cl^–^ × 1000), is recommended. This coefficient is almost constant in ocean water (139.6) (Lyman and Fleming, 1940). The data shown on Figure 5B demonstrate variation of this coefficient from 132 to 138 in the oxic zone of Lake B. Khruslomeny (to the depth of 2.50 m); these values were close to those of seawater diluted by runoff from the surface. At the depths of 3.25–4.5 m, the share of sulfate was higher than in the surface layer (144–156). This was an indication that production of additional sulfate due to light-dependent sulfide oxidation by anoxygenic phototrophic bacteria was probably more intense than sulfate consumption by sulfate reducers. The sulfate-chloride coefficient decreased to 127 at 7.0 m. Thus, the equilibrium of the sulfur balance was shifted to reductive processes, resulting in sulfate consumption and release of reduced sulfur compounds. At the lower horizons, sulfate ratio to chloride dropped sharply (to 2-5 in the near-bottom layers, which corresponded to 0.09-0.30 mmol SO_4_^2–^ L^–1^), which was an important biogeochemical result of active microbial sulfate reduction.

#### Dissolved Methane in Lake B. Khruslomeny

**FIGURE 6:**
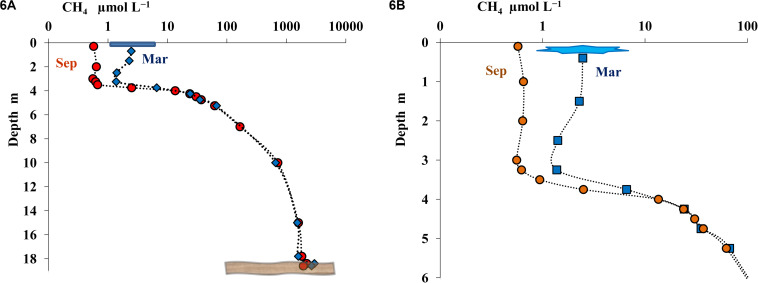
Concentration of methane (CH_4_, μmol L^– 1^) in the water column of Lake B. Khruslomeny in March (blue) and September (orange) in the water column **(A)** and in the mixolimnion and chemocline layers **(B)**.

The results of the measurements of methane distribution it the water column of Lake B. Khruslomeny in different seasons are presented on Figures 6A,B. Methane was detected throughout the water column. In September, its concentration in the upper layer (above the chemocline) was 0.2–0.6 μmol L^–1^, increased to 14–30 μmol L^–1^ in the chemocline, and then increased still further with depth, reaching a very high value of 1820 μmol CH_4_ L^–1^ in the near-bottom horizon. In winter methane was found immediately below the ice (3.2 μmol L^–1^), its concentration increased in the lower chemocline and, similar to the autumn season, was very high in the near-bottom horizon (1680 μmol L^–1^). The most abrupt change in methane concentration occurred in the horizons at 3.75–5.5 m.

#### Rates of Methane Oxidation

**FIGURE 7:**
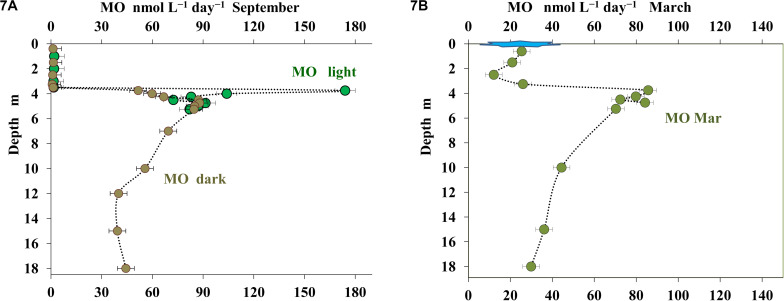
Rate of methane oxidation (MO, nmol C L^– 1^ day^– 1^) in the water column of Lake B. Khruslomeny in September: *in situ* light (green) and dark incubation (brown) in September **(A)** and *in situ* light incubation in March **(B)**.

Both in March and in September MO was shown to occur throughout the water column, from the surface to the near-bottom zone (Figures 7A,B). MO rate in the upper layer (to the depth of 3.5 m) was very low, at the limit of the sensitivity of the method (up to 1 nmol CH_4_ L^–1^ day^–1^). A rapid increase in MO rate with a peak of 100–170 nmol CH_4_ L^–1^ day^–1^ occurred at the depths from 3.75 to 4.0 m. *In situ* incubation at the depths of 3.75 and 4.0 m revealed pronounced differences in MO rates between the samples in transparent and darkened vials. Light-induced MO activation was less pronounced in the 4.25 m horizon, while no stimulation by light was observed for the samples from deeper horizons (Savvichev et al., 2019).

In March, methane oxidation was detected in all horizons, up to and including the under-ice layer. The rate of this process at depths less than 3.25 m did not exceed 26 nmol CH_4_ L^–1^ day^–1^. At depth of 3.75–5.25 m, MO rates were higher (70–85 nmol CH_4_ L^–1^ day^–1^). No experiments on MO stimulation by light were carried out in March.

### Isotope Composition of Particulate Organic Carbon, Dissolved Mineral Carbon, and Methane Carbon

**FIGURE 8:**
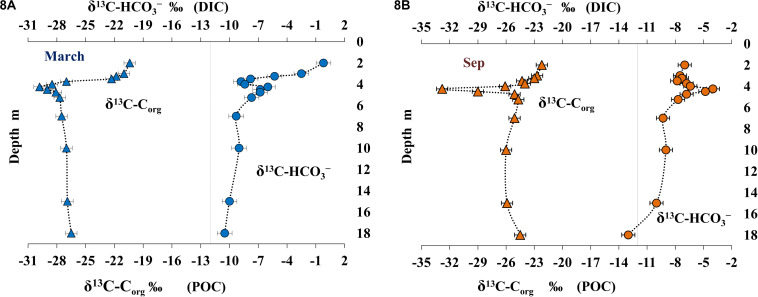
Isotope composition of particulate organic carbon (POC) (δ^13^C_org_ %) and dissolved inorganic carbon (DIC) (δ^13^C-HCO_3, –_ %) in March **(A)** and September **(B)**.

The data on isotope composition of particulate organic carbon (POC) (δ^13^C_Corg_) and dissolved mineral carbon (δ^13^C-HCO_3_^–^) are presented on Figure 8.

Carbon isotope composition of POM in the uppermost 1 m of the lake (δ^13^C_Corg_ from −21.8 to −20.4% in March and from −23.0 to −22.2% in September) was similar to the values for marine phytoplankton. Higher content of the light carbon isotope in September indicated the effect of freshwater inflow from surrounding swamped areas. The under-ice layer with a local maximum of oxygenic photosynthesis (Figure 4B) was enriched with the heavier carbon isotope (δ^13^C_Corg_ = −20.4%) from POC of newly developed autochthonous phytoplankton. Mineral carbon of the under-ice layer was enriched with the heavy isotope (δ^13^C-HCO_3_^–^ from −2.5 to −0.2%). Increased share of the heavy carbon isotope resulted from its fractionation by oxygenic phototrophs, which preferentially consume the light isotope of mineral carbon. This effect was less pronounced in September (δ^13^C-HCO_3_^–^ from −7.5 to −7.0%). In the upper water layer, the hugest efficiency of carbon isotope fractionation [Δ = (δ^13^C_Corg_ - δ^13^C-HCO_3_^–^) = 20.2%] was revealed in the under-ice water horizon. POM carbon isotope composition became significantly lighter in the upper part of the chemocline (δ^13^C_Corg_ from −29.8 to −27.0% in March and from −32.8 to −26.1% in September; Figures 8A,B). The carbon isotope composition of carbonate and CO_2_ also changed within this horizon (δ^13^C-HCO_3_^–^ = −6.0% in March and −4.0% in September; Figures 8A,B). Changes in the composition of mineral carbon resulted from two processes: CO_2_ release due to oxidation of methane arriving from deeper layers and CO_2_ consumption by anoxygenic photosynthesis. As a product of methanotrophic activity, the carbon of carbonates and CO_2_ inherits the lighter isotopic composition of methane. The newly produced light mineral carbon is in turn consumed by the anoxygenic phototrophic bacterial community and incorporates into organic matter produced in this layer with high microbial activity. The δ^13^C_Corg_ in the deeper anoxic layer varied from −27.5 to −26.5% in March and from −26.0 to −24.5% in September. The mineral carbon was enriched with the light isotope (δ^13^C-HCO_3_^–^ varied from −13.0 to −9.0%). Lighter isotope composition of mineral carbon in the near-bottom horizons resulted from intense processes of anaerobic degradation of OM produced by the community of anoxygenic phototrophs, chemotrophs, and methanotrophs and arriving from higher water layers. The efficiency of carbon isotope fractionation in the near-bottom anoxic layer was lower than in the chemocline and the subsurface horizons (Δ = 16-18% in March and 12–17% in September).

**FIGURE 9:**
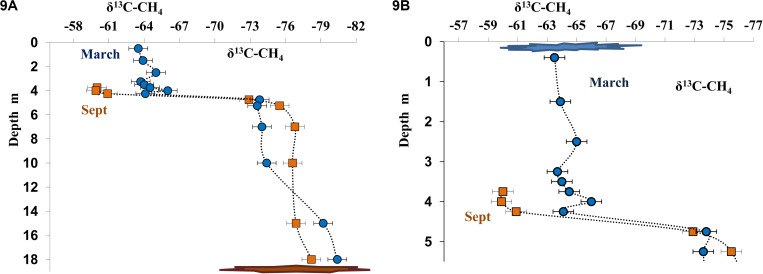
Isotope composition of methane carbon (δ^13^C-CH_4_, %) in the water column of Lake B. Khruslomeny in March (blue) and September 2017 (orange) in the water column **(A)** and in the chemocline and mixolimnion layers **(B)**.

Dissolved methane from the near-bottom water, where its concentration was very high both in March and September, was isotopically light (δ^13^C_CH__4_ from −78.0 to −80.4%, Figures 9A,B). The content of isotopically light methane carbon decreased in higher horizons, including the chemocline (δ^13^C_CH__4_ from −64.0 to −60.0%; Figure 9) due to its microbial consumption (oxidation). In September, carbon isotope composition of dissolved methane was determined for the layers from the bottom to 3.75 m (Δ^13^C_CH__4_ = −60.0%). The concentration of dissolved methane in the upper layers was insufficient for analysis during this season. The data on methane carbon isotope composition in the three horizons above the chemocline (δ^13^C_CH__4_ from −65.0 to −63.5%) were obtained in March (Figure 9B). A significant increase in the content of the heavier carbon isotope indicated fractionation resulting in the course of microbial (aerobic) methane oxidation.

### Microbial Community of the Water Column of Lake B. Khruslomeny

**FIGURE 10:**
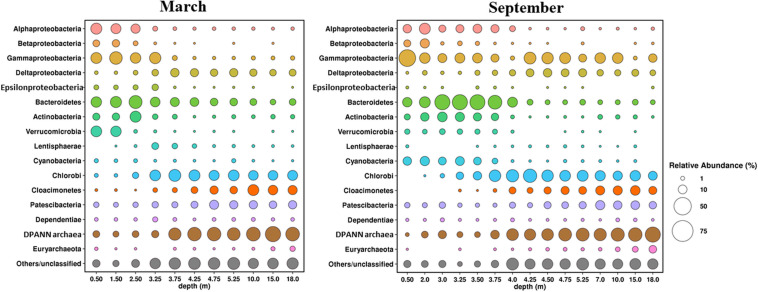
Composition of microbial communities in water column of Lake B. Khruslomeny in March and September determined by high-throughput sequencing of the 16S rRNA genes.

Microbial communities of the water column of the lake in March and September 2017 (11 horizons in March and 15 in September) were characterized by analyzing their 16S rRNA gene sequences (Figure 10).

#### Oxic Zone

Pro- and eukaryotic microorganisms carrying out oxygenic photosynthesis were the primary producers in the oxic upper layer of the lake. The composition of the phototrophic community in September and March was found to differ significantly. Cyanobacteria predominated (7 to 12% of the community) in the upper horizon (0–3.35 m) in September. Almost all of them belonged to the genus *Cyanobium* of the order Synechococcales, which is common in Arctic basins (Van Hove et al., 2008). During the ice-covered season the share of cyanobacteria in the uppermost water horizon was ∼1%, probably due to decreased illumination. The most numerous bacterial groups in the upper layer were Alphaproteobacteria (6–18%), Gammaproteobacteria (11–46%), Bacteroidetes (12–36%), Actinobacteria (4–13%), and Verrucomicrobia (2–3%). Cultured members of most of these groups are aerobic heterotrophs. Thus, predominant Alphaproteobacteria belonged to Rhodobacteraceae and Hyphomonadaceae, including members of the genus *Hyphomonas*, common in marine environments, including the Arctic seas (Li et al., 2014), as well as to the uncultured clade SAR11, one of the most abundant cosmopolitan lineages of marine plankton (Giovannoni, 2017). Predominant members of the Gammaproteobacteria belonged to the genera *Aeromon*as, *Shewanella*, and *Psychrobacter*, while in the uppermost 0.5-m layer, the genus *Pseudomonas* prevailed, constituting ∼20% of the community. The Bacteroidetes was one of the most diverse microbial groups containing 183 OTUs, mainly belonging to the orders Chitinophagales, Sphingobacteriales, and Flavobacteriales, as well as to the uncultured lineage VC2.1 Bac22. All these groups comprise typical aquatic heterotrophs. Predominant actinobacteria were *Candidatus* Aquiluna (family Microbacteriaceae), actinorhodopsin-carrying photoheterotrophs isolated from marine and freshwater environments (Kang et al., 2012). Betaproteobacteria were numerous (6–11%) only in the desalinated upper layer at the depths not exceeding 2 m, while deeper their share was only 1–2%. Most of the Betaproteobacteria belonged to two groups of the family Burkholderiaceae, the genus *Limnohabitans*, comprising freshwater aerobic anoxygenic phototrophic bacteria, which were found in pelagic zones of various freshwater habitats (Kasalický et al., 2018), and an uncultured MWH-UniP1 aquatic group. The same bacterial phyla, except for Cyanobacteria, were predominant in the oxic upper zone during winter. The share of Verrucomicrobia in the desalinated upper layer at 0.5–1.5 m was significantly higher (16–17%) due to abundance of three OTUs, two of which belonged to the genus *Luteolibacter* (family Rubritaleaceae), while the third belonged to the genus *Prosthecobacter*. Members of these genera have been isolated from diverse aquatic ecosystems, including Arctic soils and lakes (Jiang et al., 2012). Some strains were shown to degrade the components of algal cells (Ohshiro et al., 2012; Lee et al., 2014). Active algal growth in the under-ice layer probably favored development of these verrucomicrobia.

#### Chemocline

Green sulfur bacteria of the phylum Chlorobi capable of anoxygenic photosynthesis constituted ∼20% of the bacterial community both in September and in March. They were almost exclusively represented by a single OTU, which was identified as *Chlorobium phaeovibrioides.* Members of this species can form very dense colored layers in the water, acting as bacterial filters preventing sulfide release from the monimolimnion to the epilimnion (Savvichev et al., 2018). *C. phaeovibrioides* has been found in the chemocline and hypolimnion of meromictic basins at the Kandalaksha coast, White Sea. Metagenomic analysis of the water sample from the chemocline of Lake B. Khruslomeny collected in March 2017 revealed the same species, which was responsible for ∼25% of the metagenome (Kadnikov et al., 2019a). In September, the dominant groups in the chemocline zone (3.5–3.75 m) included Bacteroidetes (30–36%), Alphaproteobacteria (8–10%), Gammaproteobacteria (8–13%), Actinobacteria (7–8%), Cyanobacteria (3–7%), Deltaproteobacteria (2–3%), Verrucomicrobia (∼2%), and archaea of the candidate phylum Woesearchaeia (5–14%). The lineages found among the Bacteroidetes, Gammaproteobacteria, Cyanobacteria, Actinobacteria, and Verrucomicrobia were mainly the same which were detected in the upper layer. Among the *Alphaproteobacteria*, the main components of the community were Rhodobacteraceae and Magnetospiraceae, while Hyphomonadaceae and SAR11, which were present in the upper oxic zone, were not found. In the upper part of the chemocline (3.5–3.75 m), where methane oxidation was active (Figure 7A), members of the genus *Methyloprofundus* (Methylomonaceae, Gammaproteobacteria) were revealed; these organisms have been isolated from marine sediments and water column (Tavormina et al., 2015). Their share at 3.75 m was as high as 2.4%. These bacteria were nearly absent in September samples from the upper oxic zone.

In winter the chemocline was closer to the surface, at the depths of 2.5–3.25 m. While the composition of the chemocline microbial community in March was mostly similar to that observed in September, some significant differences were found. First, the share of cyanobacteria in the chemocline zone was lower, not exceeding 1%. Second, in winter methanotrophs occupied higher horizons, including the under-ice layer (0.5–3.25 m), and their share in the community at 1.5 m was as high as 14%. Apart from *Methyloprofundus*, *Methylobacter* was also present in the methanotrophic community. Members of this genus have been isolated from Arctic swamp soils, Svalbard (Wartiainen et al., 2006; Kallistova et al., 2014). Third, members of the genus *Thiomicrorhabdus*, chemolithotrophic gammaproteobacteria oxidizing reduced sulfur compounds at low levels of oxygen (Boden et al., 2017), were found in the chemocline. At the depths of 2.5 and 3.32 m the shares of *Thiomicrorhabdus* were 7 and 18%, respectively. These organisms were not revealed in September samples, and in March their share in other horizons did not exceed 1.5%. Another group of chemolithoautotrophic sulfur-oxidizing bacteria, members of the genus *Sulfurimonas* (Epsilonproteobacteria), was also revealed in the chemocline and above. Their share was 1.7 to 4.5% at the depths of 0.5–3.25 m and less than 0.2% at greater depths; in September, they occurred in minor amounts (0.5%) only in the chemocline (3.25 m). The winter samples also contained high numbers of bacteria of the phylum Lentisphaerae in the lower chemocline zone (3.25 and 3.75 m).

#### Anoxic Zone

The hypoliminion microbial communities (samples collected from the depths exceeding 4.25 m in September and 3.75 m in March) differed in composition from those of the upper oxic zone and the chemocline, while the differences between the March and September samples were, barring some exceptions, less pronounced. The five groups most numerous in September were Chlorobi (10–27%), Gammaproteobacteria (1–21%), Cloacimonetes (4–13%), Deltaproteobacteria (3–9%), and Patescibacteria (2–11%). The shares of other bacterial phyla did not exceed 1%. Over 90% of the sequences of the most numerous phylum, Chlorobi, belonged to a single species *Chlorobium phaeovibrioides*.

The phylum *Candidatus* Cloacimonetes (previously known as candidate division WWE1) was originally observed in anaerobic digesters (Solli et al., 2014). Analysis of the genomes of these microorganisms revealed their ability to utilize proteinaceous substrates (Pelletier et al., 2008), cellulose (Limam et al., 2014), and propionate (Nobu et al., 2015), as well as to form syntrophic associations with other microorganisms (Dyksma and Gallert, 2019). Cloacimonetes were revealed in various anaerobic ecosystems, including the Black Sea sulfide zone, where they were found to be responsible for degradation of dissolved organic matter (Suominen et al., 2019). The highest share of Cloacimonetes (8–13%) occurred at the depths below 7 m. The closest relatives have been found in the sediments of meromictic lakes and in Pacific hydrothermal vents.

Most of the Deltaproteobacteria belonged to Desulfarculaceae and Desulfobacteraceae; cultured members of these families are sulfate reducers. The most numerous OTU was assigned to the genus *Desulfatiglans*. Characterized *Desulfatiglans* isolates are dissimilatory sulfate reducers capable of degrading aromatic compounds (Suzuki et al., 2014), while *Desulfatiglans*-related microorganisms found in marine sediments could be metabolically more diverse: apart from sulfate reduction, some of them exhibited the genetic potential for growth by acetogenesis, fermentation, and reductive dehalogenation (Jochum et al., 2018). Members of the family Syntrophaceae were also found at the depths below 7 m. Cultured members of this family are syntrophs degrading short-chain fatty and aromatic acids to produce acetate, formate, and hydrogen in co-cultures with hydrogen-consuming methanogens or sulfate reducers (Elshahed and McInerney, 2001; McInerney et al., 2007).

Gammaproteobacteria constituted 8 to 21% of the microbial community in all samples below the chemocline, except for 15 m where their share was only 0.92%. A single OTU assigned to the genus *Pseudoalteromon*as, which was numerous in the chemocline zone as well, was responsible for this heterogeneity. *Pseudoalteromonas* species are a group of mostly aerobic marine bacteria frequently found in various environments, including cold habitats and deep-sea sediments (Parrilli et al., 2019). In the samples collected from the depths of 0.5, 2, 3, 4, and 15 m in September, as well as in all March samples, the share of this OTU did not exceed 1%. Since *Pseudoalteromonas* species are generally found in association with marine eukaryotes (Holmström and Kjelleberg, 1999), detected *Pseudoalteromonas* were probably also associated with macroscopic objects accidentally collected with the samples.

Members of the superphylum Patescibacteria, also known as the candidate phyla radiation (CPR), a large monophyletic group in the tree of life, which lacks cultivated representatives, constituted a significant part of the hypolimnion microbial community (2 to 12%). Members of the Patescibacteria group have been found in geothermal pools, marine and freshwater sediments, soil, and other mostly anoxic organic-rich environments. Genomics studies of Patescibacteria revealed that they have small genomes (∼1 Mbp or less) with limited capacities for fermentative metabolism, and lacked amino acid, nucleotide, and lipid biosynthetic pathways, which suggests the lifestyle of a scavenger or symbiot/parasit (Brown et al., 2015; Castelle et al., 2018).

Both in March and September archaea constituted from one-third to a half of all detected microorganisms in the hypolimnion samples. The putative methanogens constituted a small part of the archaeal community and occurred mostly in the samples of near-bottom water (depths of 15 and 18 m), where their shares among all 16S rRNA gene sequences were 0.9 and 2% in March and 3 and 6% in September, respectively. The total share of methanogenic archaea in other samples did not exceed 0.5%. Most methanogens belonged to hydrogenotrophic archaea of the order Methanomicrobiales; aceticlastic methanogens of the family Methanosaetaceae (mostly the genus *Methanosaeta*) and methylotrophic methanogens of the family Methermicoccaceae were also revealed. Notably, we found no 16S rRNA gene sequences related to known anaerobic methane oxidizers (ANME-1, 2, and 3).

Members of the DPANN superphylum, mainly Woesearchaeota, were the predominant archaeal group responsible for up to 50% of all 16S rRNA gene sequences. Members of the DPANN superphylum (Rinke et al., 2013) are widespread in various aquatic ecosystems, including boreal and subarctic lakes (Restrepo-Ortiz and Casamayor, 2013; Ortiz-Alvarez and Casamayor, 2016; Carnevali et al., 2018; Kadnikov et al., 2019b; Kallistova et al., 2019).

## Discussion

Since microbial communities developing in the chemocline of meromictic lakes carry out geochemically important redox processes, such basins are of interest to microbiologists. As a rule, two layers of photosynthetic activity are present in meromictic lakes: the layer of oxygenic photosynthesis in the surface or subsurface horizon (meters to several tens of meters thick) and the layer of anoxygenic photosynthesis, which usually coincides with the chemocline (oxycline) and is one to several meters thick. As a rule, oxygenic photosynthetic production in meromictic lakes exceeds the anoxygenic one, since the community of anoxygenic phototrophic bacteria relies on residual sunlight penetrating the upper water layers inhabited by cyanobacteria and algae. If the chemocline horizon is located in the photic zone, anoxygenic phototrophic bacteria in high or very high numbers constitute the basis of the chemocline microbial community. Dense microbial layers, easily discernible due to their green or red pigmentation, are sometimes termed bacterial plates (Vila et al., 1998; Garcia-Gil et al., 1999; Camacho et al., 2001).

Meromictic basins (lakes, ponds, bays, and fjords) are known on all continents and many islands (Zadereev et al., 2017). They vary widely in size, from 1 acre to the area of the Black Sea, the largest meromictic basin (Pimenov and Neretin, 2006). While chemocline is present in all meromictic basins, the density (abundance) of the microbial community may vary widely. Lake Mahoney (Canada), one of the lakes best studied from the microbiological point of view, probably has the highest density of the chemocline community. Abundance of purple sulfur bacteria in its dense layer was shown to exceed 10^8^ cells mL^–1^ (Overmann, 1997). Metagenomic analysis of the community of the bacterial plate in the chemocline layer revealed purple sulfur bacteria of the genus *Thiohalocapsa* (family Chromatiaceae) to be the main primary producers (Hamilton et al., 2014). In Lake Khruslomeny, this function was performed by green sulfur bacteria *Chlorobium phaeovibrioides*, which was probably due to the differences in water composition between these two lakes. Lake Mahoney is saline and alkaline, with the salt content of its water differing from that of seawater.

A meromictic Lake Suigetsu (Wakasa Bay, Sea of Japan) is similar to the basins of the Kandalaksha Bay coast. This lake has a limited connection to the sea bay. Analysis of the pigments from Lake Suigetsu water column revealed predominance of bacteriochlorophyll *e*, originating from brown-colored green sulfur bacteria (Kondo et al., 2014). The numbers of phototrophic bacteria in the chemocline exceeded 5 × 10^6^ cells mL^–1^ (June). Analysis by quantitative real-time PCR revealed that green sulfur bacteria *Chl. phaeovibrioides*, *Chl. limicola*, and *Chl. luteolum* were predominant in the chemocline microbial community (Mori et al., 2013). This is an indication of the similarity between microbial communities of the lakes Suigetsu and B. Khruslomeny.

A meromictic Lake Nitinat (British Columbia, Canada), located in a fjord bed (Schmidtova et al., 2009), is geochemically similar to Lake B. Khruslomeny. Location of its chemocline (transition zone) depends on the season. In April, *Chlorobium* species dominated the chemocline library, including two highly represented clusters: one related to the environmental clone *Chlorobium* sp. Mog 4 (EF149015) from Lake Mogilnoe (Lunina et al., 2005) and the other related to *Chl. phaeobacteriodes* BS1 (CP001101) isolated from the Black Sea chemocline (Manske et al., 2005) *Chlorobium* species were accompanied by epsilonproteobacteria most closely related to thiotrophic endosymbionts of marine invertebrates, as well as by *Arcobacter* sp., which was capable of growth at high sulfide concentrations and traces of oxygen (1–10 μM). Two sequences were related to a methanotrophic endosymbiont of *Bathymodiolus* sp., and several others also clustered together within the Methylococcales (Schmidtova et al., 2009).

The meromictic Clipperton lagoon is located in the tropical Pacific, to the southwest from the Mexican coast. The lagoon became separated from the ocean ∼160 years ago. Its greatest depth is 45 m, and it has a classical meromictic profile of the water column, with a saltish mixolimnion and an anoxic monimolimnion at almost marine salinity, which are separated by the chemocline at 13–18 m. Green sulfur bacteria (Chlorobi) predominated in the chemocline microbial community, with their numbers up to 6 × 10^6^ cells mL^–1^. Only two different genera of green sulfur bacteria were predominant in the libraries: *Prosthecochloris* and *Chlorobium*. Analysis of the *pmoA* functional gene in the pycnocline revealed occurrence of methanotrophs (genera *Methylomonas* and *Methylococcus*). The gene indicative of sulfate reduction, *dsrAB*, could only be amplified from the pycnocline. All sequences belonged to the Deltaproteobacteria. The *dsrAB* genes were not detected in the monimolimnion, even though the 16S rRNA gene sequences from typical sulfate-reducing Deltaproteobacteria were present.

In the relic Lake Mogilnoe (Kildin Island, Barents Sea), which is also connected to the sea, anoxygenic green phototrophic bacteria *Chl. phaeovibrioides*, *Pelodictyon phaeum*, and *Prosthecochloris phaeoasteroidea* formed the backbone of the chemocline microbial community. Purple sulfur bacteria *Thiocapsa roseopersicina* and *Thiocystis gelatinosa* were the minor components (Lunina et al., 2005).

The present work dealt with a meromictic lake recently separated from a sea bay and retaining the salt composition similar to that of seawater. Similar to Lake B. Khruslomeny, in Lake Trekhtsvetnoe, the subject of our previous study, the phototrophic community of the dense layer was represented exclusively by green sulfur bacteria *Chl. phaeovibrioides* (Savvichev et al., 2018). The numbers of these microorganisms in this narrow layer (bacterial plate were as high as 2.1 × 10^7^ cells mL^–1^.

Among the Kandalaksha Bay coastal basins with limited connection to the sea, Lake B. Khruslomeny is characterized by its stable hydrological regime. Its stable stratification is probably the factor responsible for formation of the dense green layer. Lake B. Khruslomeny chemocline is characterized by the evident predominance of *Chl. phaeovibrioides*, extremely high rate of anoxygenic photosynthesis (up to 34.5 μmol C L^–1^ day^–1^), high rate of light-stimulated methane oxidation (up to 170 nmol CH_4_ L^–1^ day^–1^), and occurrence of *Synechocystis* cyanobacteria and *Methyloprofundus* methanotrophic gammaproteobacteria.

Methane content in the Lake B. Khruslomeny monimolimnion (from 1.68 to 1.82 mmol CH_4_ L^–1^; Figure 6) was stable and very high, comparable to the values for the known meromictic basins: deep-water Lake Matano in Indonesia (Crowe et al., 2008), Lake Kivu in Eastern Africa (Schmid et al., 2005), and Lake Pavin in France (Lehours et al., 2005). Considering the total depth of the lake, the calculated methane content in the water column was 12.5 mol CH_4_ m^–2^ in September and 12.2 mol CH_4_ m^–2^ in March. Over 99% of methane was concentrated in the monimolimnion. Methane was most actively consumed in the chemocline at the rate of up to 175 nmol CH_4_ L^–1^ day^–1^ (September).

In September, methane oxidation was most intense in the narrow depth interval from 3.75 to 4.0 m; no noticeable stimulation of this process by light was observed there. The zone where light-dependent methane oxidation occurred was at the lowermost horizon where oxygen measurement was possible. Methanotrophic gammaproteobacteria of the family Methylococcaceae were detected in the same depth interval. In this horizon (3.75 m), cyanobacteria of the order Synechococcales were also revealed; moreover, the share of their 16S rRNA gene sequences (2.5–3%) was close to that of methanotrophs. The results of radiotracer studies and molecular analysis indicated light-dependent methane oxidation, which was most probably carried out by methanotrophic gammaproteobacteria in association with cyanobacteria, which provide oxygen for this process (Savvichev et al., 2018). According to the equation CH_4_ + 2O_2_ = CO_2_ + 2H_2_O, the calculated oxygen requirement for MO at 3.75 and 4.0 m is 0.35 and 0.11 μmol O_2_ L^–1^ day^–1^, respectively. The calculated oxygen production via oxygenic photosynthesis (CO_2_ + H_2_O = CH_2_O + O_2_) is 1.18 and 0.40 μmol O_2_ L^–1^ day^–1^ at 3.75 and 4.0 m, respectively. This is sufficient for methane oxidation. Results of such calculations should, be treated with caution, since, apart from methane, oxygen may react with other reduced compounds.

Light-dependent methane oxidation has been previously reported only for freshwater meromictic lakes from diverse climatic zones (Milucka et al., 2015; Oswald et al., 2015, Oswald et al., 2016; Kallistova et al., 2019), where methanotrophic gammaproteobacteria were detected in the presence of analytically measurable oxygen concentrations. In the chemocline of Lake B. Khruslomeny salinity was approximately one-third of that of seawater. Therefore, while methanotrophs of the genus *Ìethylobacter* predominate in freshwater continental meromictic lakes (Kallistova et al., 2019), typical marine methane-oxidizers of the genus *Methyloprofundus* prevailed in the Lake B. Khruslomeny chemocline in September. The composition of the hypolimnion water column of the lake was of the marine type. During winter, aerobic methanotrophs (both *Methyloprofundus* and freshwater *Methylobacter*) were revealed mainly in the upper oxic layer (0.5–1.5 m), above the chemocline, where cyanobacteria were also present (∼1%). Thus, methane oxidation occurred in winter mainly in the upper oxic zone, rather than in the chemocline, and was less dependent on activity of cyanobacteria. High numbers of chemolithotrophic sulfur-oxidizing bacteria of the genera *Thiomicrorhabdus* and *Sulfurimonas* were also present in the chemocline and upper horizons during winter. Lower illumination and photosynthetic activity in winter probably prevented *Chl. phaeovibrioides* from utilizing all sulfide produced by sulfate reducers, so other sulfur oxidizers could develop as well.

In the presence of dissolved sulfate, the possibility of sulfate-dependent methane oxidation in the anoxic water column could not be ruled out. However, no 16S rRNA gene sequences typical of ANME archaea were detected. Likewise, the NC10 bacteria oxidizing methane with nitrite were also not detected in the chemocline or hypolimnion. Thus, the present work shows that light-dependent methane oxidation is not restricted to continental freshwater stratified lakes, but may contribute significantly to the oxidation of this important greenhouse gas in the sea coastal reservoirs, which were separated from the major marine basin, but retained partial connection to it.

Lake B. Khruslomeny is a lake where microorganisms formed a complex community with predominance of Chlorobi green sulfur bacteria in the chemocline, which was capable of efficient utilization of reduced compounds (sulfide and methane) on their way from the bottom sediments to the surface.

Interestingly, one-third to a half of the microorganisms from the oxic zone belonged to uncultured bacterial and archaeal lineages, the superphyla Patescibacteria and DPANN, with Woesearchaeota constituting the major share of all uncultured groups. These two microbial groups both have small cells (<1 μm) and small genomes; they are considered to possess limited metabolic abilities, to be incapable of oxidative metabolism, and to lack a number of pathways for biosynthesis of the key cell components, which may indicate their partner-dependent (symbiotic or parasitic) lifestyle (Castelle et al., 2015, 2018). Particularly, recent abundance distribution and co-occurrence network analyses across diverse biotopes suggested a potential syntrophic relationship between Woesearchaeota and methanogens (Liu et al., 2018). However, the share of methanogenic archaea in Lake B. Khruslomeny is low, and their distribution with depth differs from that of Woesearchaeota. High occurrence of DPANN archaea, especially Woesearchaeota, in freshwater lakes has been reported in several studies (e.g., Bowman et al., 2013; Kallistova et al., 2019). Abundance of these organisms indicates that they may play an important ecological role. Thus, it was recently shown that Woesearchaeota and Patescibacteria form an important part of the protein-degrading microbial communities in the Black Sea anoxic waters (Suominen et al., 2019). Moreover, due to a higher ratio of surface area to volume, small cell size facilitates nutrient uptake and increases the metabolic rate (Savage et al., 2007). Therefore the role of scavengers efficiently utilizing organic compounds and metabolites could be proposed for Patescibacteria and DPANN in the Lake B. Khruslomeny.

## Data Availability Statement

The 16S rRNA gene sequences were deposited in the Sequence Read Archive (SRA) via the National Center for Biotechnology Information (NCBI) under accession nos. SRR11816416–SRR11816441.

## Author Contributions

AS, NR and NP contributed to the conception and design of the study. AS, VK, IR, EK, DV, NK, GL, ND, and NB participated in field research and initial treatment of the materials. VK, AB, AM, and NR carried out molecular ecological and bioinformatic analysis. AK and PS contributed to the writing and editing of the manuscript. All authors contributed to the article and approved the submitted version.

## Conflict of Interest

The authors declare that the research was conducted in the absence of any commercial or financial relationships that could be construed as a potential conflict of interest.
